# Knowledge and perceptions of hepatitis c infection and pesticides use in two rural villages in Egypt

**DOI:** 10.1186/1471-2458-14-501

**Published:** 2014-05-24

**Authors:** Doa’a A Saleh, Sania Amr, Irene A Jillson, Judy Huei-yu Wang, Walaa A Khairy, Christopher A Loffredo

**Affiliations:** 1 Cairo University, Cairo, Egypt; 2 University of Maryland, Baltimore, MD, USA; 3 Lombardi Cancer Center, Georgetown University, 3970 Reservoir Rd, Washington, DC 20057, USA

**Keywords:** HCV, Pesticides, Liver cancer, Hepatocellular carcinoma, Knowledge

## Abstract

**Background:**

Hepatocellular carcinoma (HCC), one of the most fatal types of malignancy, is increasing worldwide, and particularly in Egypt where there is a confluence of its contributing factors, including high prevalence of hepatitis C virus (HCV) infection, widespread use of pesticides, and diets that are contaminated by aflatoxin B1 (AFB1) in rural areas. We investigated knowledge, attitudes, and prevention practices related to HCV infection and pesticides use in rural Egypt, where over half of the population resides and agriculture is the predominant occupation.

**Methods:**

From two rural villages we recruited 67 residents aged 18–80 years, who completed a 40-item survey that included questions about demographics, knowledge of and protective measures relevant to pesticides use in the home and in agriculture, awareness and perceptions of HCV infection and its treatment and prevention.

**Results:**

Among the 67 study participants, gender distribution was equal, the mean age was 47.2, and one third never attended school. More than 50% reported using pesticides at home, but fewer reported having some knowledge about its health effects. Twelve participants were agricultural workers, and 11 of them applied pesticides in the field and knew about their toxicity; however only one person was correctly using the appropriate protective equipment. Among all the participants, 52 did not know what causes HCV infection, and 42 of those who knew it was a virus mentioned incorrect modes of transmission; and 30 did not know the disease manifestations.

**Conclusion:**

In rural Egypt, there is a significant lack of knowledge of HCV infection and its transmission mode and limited use of protective measures against pesticides despite familiarity with these chemicals.

## Background

Hepatocellular carcinoma (HCC), primary liver cell cancer, one of the most prevalent and fatal types of malignancy, is increasing worldwide, and particularly in Egypt. Among the well-established factors that contribute to the development of HCC, chronic infection with hepatitis C virus (HCV) has emerged as the primary cause in the USA, Europe, the Middle East, and North Africa [1-3]. Indeed, during the past two decades of epidemiological research in Egypt [4-10], we and others have observed that the major risk factors for HCC are chronic infections with HCV and hepatitis B virus (HBV), along with occupational exposures to pesticides and contamination of the diet with aflatoxin B1.

Egypt suffers from the world’s highest prevalence of HCV infection - up to 24% in rural areas [9] - and intra-familial spread of HCV has been documented as one of the most important routes of transmission [11,12] leading to the current estimated burden of 10 million infected Egyptians. Added to this burden is the widespread use of pesticides as well as diets that are contaminated by aflatoxin B1 in rural areas, where over half of the Egyptian population resides and agriculture is the predominant occupation. Additionally, the standard treatment for HCV infection, which consists of combination therapy with pegylated interferon and Ribavirin, has serious side effects and its cure rate is limited to 25-70%, depending on the HCV genotype. Moreover, it is very expensive, consequently precluding most patients in developing countries from access. Given the large reservoir of HCV in Egypt and the country’s high fertility rate when compared to developed countries, its society faces an increasing burden of patients who acquire the virus in childhood or young adulthood, placing them at greater risk of developing HCC later in their lives.

Besides the lack of vaccine to prevent infection, a global challenge to managing HCV is the lack of a prevention model that addresses the issue at the community level. Therefore, we conducted a pilot study in two rural villages to investigate knowledge and prevention practices related to HCV and pesticides use; both of which are significant risk factors for HCC in rural Egypt. We used a survey to collect information that can be used to inform future prevention intervention designs.

## Methods

### Study sample and recruitment

The study protocol was approved by the Institutional Review Boards of Georgetown University and the National Cancer Institute of Cairo University, and participants were recruited from two rural villages in the Menoufia and Giza governorates of Egypt. Eligible study participants included those who were village residents aged 18–80 years old and self-identified as capable of completing a 20 minute interview; that is, without a physical or mental health disability that would hinder participation in an interview.

On a given day, the study recruiters visited the selected villages, where a central street was randomly selected from the village map and a systematic random sampling method was applied to approach residents on both sides of the street. If none of the house occupants matched the eligibility criteria, the recruitment team moved to the next house. When all households had been contacted on the street, the team turned right at the nearest intersection and began approaching houses on the new street. Once a potentially eligible participant was identified, the trained interviewer explained the study to the prospective subject and offered participation. Those who agreed to participate were asked to read and sign an informed consent document, with a witness signing the forms for illiterate participants who verbally agreed to participate. Information that could potentially be used to identify individuals was deleted from the data collection instruments.

### Data collection instrument

Based on a review of the literature [13-16] and experience in the field, the study team developed a 40-item semi-structured survey tool, including both open- and closed-ended questions (See Additional files 1 and 2 for the Arabic questionnaire and its English translation). The tool comprised four sections: 1) demographics; 2) knowledge of and perceptions regarding pesticides used in the home and in agriculture; 3) knowledge and awareness of HCV; and 4) perceptions of prevention and treatment of HCV.

### Data analysis

For the close-ended questions, descriptive analysis was carried out to calculate mean and standard deviation and frequency and percentage for continuous and categorical variables, respectively, using Statistical Package for the Social Sciences (SPSS) software (version 15.0). The open-ended questions were translated from Arabic to English and reviewed by two of the authors.

## Results

### Demographic characteristics

Between April and May, 2011, we approached approximately 75 residents; we were able to recruit 67 (40 from Menoufia and 27 from Giza) who agreed to participate and complete the survey, 34 of whom were female (51%) and 33 (49%) of whom were male. The mean age ± standard deviation (SD) was 47.2 ± 12.4 years (median 45). Nearly one third never attended school, 18% attended only either literacy classes or primary school, and 40% attended preparatory, secondary or technical school. Only 8% reported having attended university. Reported illiteracy was common: 28% reported that they could not read, and 18% indicated that they could read with difficulty.

Three-fourths of respondents (N = 50) reported working outside the home, of whom 90% worked for cash. Twelve were farmers (11 from Menoufia and only one from Giza) and five worked in health and social services. Most respondents (93%) reported that they used municipal water for drinking. About two-thirds (60%) had an indoor latrine, and about 40% had a flush toilet.

### Knowledge and perceptions of pesticides used at home

More women (74%) than men (48%) and more residents from Menoufia (68%) than from Giza (48%) reported using pesticides or rodenticides at home; however, members of both genders were unaware of the adverse health effects of pesticides use (62% versus 61%, among women and men, respectively). Four respondents did not know the specific types they used, but all others provided information about the name or formulation of the products. Of these, 18 reported using insecticidal sprays for flies and mosquitoes and mentioned some commercial brand names available in Egypt; six reported using powder or dough for ants and cockroaches without specifying the products’ names. With regard to rodenticides, 13 participants reported using some products but did not specify their names.

When the participants were asked what types of health conditions are related to pesticides used at home, almost one fifth of participants (19%) said they did not know. Fifty-four participants (81%) mentioned one or more health effects (Table 1) - toxicity (43%) and allergy (25%) being the most reported ones. Very few participants mentioned that the use of pesticides can cause health problems such as drowsiness, gastrointestinal problems, cancer, and death. Only two participants mentioned that it can affect children and another two said that the health effects depend on the type of pesticides used.

**Table 1:** Adverse health effects of and protective measures for pesticides use at home

	**N (total = 67)**	%*
**Adverse health effects**		
Toxicity	29	43
Allergy	17	25
Skin reaction	12	18
Respiratory symptoms	12	18
Asphyxia	6	6
Eye irritation	4	9
Don’t know	17	25
**Protective measures**		
Leaving the sprayed room and close it after spraying	17	25
Using masks that cover mouth and nose	11	17
Using gloves	9	13
Washing hands after spraying	6	9
Keeping away from food or drink	5	8
Keeping away from children	4	6
Don’t know	8	12

*Percents may add to more than 100% due to multiple responses.

When asked if they were ever told about possible adverse health effects following the use of pesticides at home, only 26 participants answered yes. Of these, eleven had been informed by a health provider, agricultural extension worker, or pesticides vendor. Other sources of warnings included relatives or friends (15%), pamphlets (15%), and television and mass media (8%). Five participants reported that the harmful effects of pesticides were “general knowledge”.

With regards to the protective measures that should be followed during spraying of pesticides, participants mentioned one or more protective measures (Table 1). Closing and leaving the sprayed room was the most mentioned measure (25%) followed by using masks (16%). Eight participants did not know how to prevent pesticide exposure, while others mentioned keeping the chemicals away from children and food.

### Knowledge and perceptions of pesticides used in agriculture

Of the 67 respondents, 12 reported agriculture as their primary occupation. Of these, all but one used pesticides in their agricultural field activities. Seven of these 11 participants used pesticides only once a year, while four used them three or more times a year. When asked about the names of pesticides they had been using, six stated that the specific product depended on the type of crop. Four of 11 respondents mentioned using malathione, while three reported using rodenticides without specifying the brand names. Three other farmers mentioned using unspecified herbicides to control weeds.

With respect to the adverse health effects of agricultural pesticides, ten of the 11 participants stated that toxicity was the most significant detriment, followed by allergy and skin manifestations (36% each) and different types of respiratory symptoms (18%). Other types of adverse health effects were mentioned by two or fewer respondents (Table 1).

All 11 agricultural pesticides users reported that they had been informed about the health problems related to pesticides. Of these, six had been informed by agricultural extension workers, three had been informed by community leaders, two by mass media and one reported reading the pamphlet available with the pesticide product. Nine participants reported having been told how to protect themselves from exposure to pesticides. When asked about these protective measures, three participants did not specify the measures and said to “*just be cautious during spraying*”; four others mentioned that they had been advised to use masks, two to use gloves, and two discussed the importance of using the appropriate amount of pesticide. Other responses such as washing hands after spraying, avoiding contact with the eyes, spraying in the direction of the wind, and not eating or drinking in the sprayed field were each mentioned by one participant. When asked about the protective measures they actually used, six reported using masks and six reported using gloves; just four reported washing their hands, and only one reported using protective clothing or coverings while spraying, or taking a shower after spraying. When the farmers were asked whether they had ever experienced adverse health effects related to the use of agricultural pesticides, only one participant reported suffering from dermatitis and itching, for which he sought medical advice from a pharmacist.

### Knowledge and awareness of HCV

The results of the interviews revealed that a large number of respondents did not know the causes or had misconceptions regarding HCV infection. Of the 67 total participants, 22% (seven females and 8 males; 10 from Giza and 5 from Menoufia) stated that they did not know the cause of HCV infection. All the other 52 subjects mentioned one or more modes of transmission, yet among them most (81%) mentioned incorrect modes such as polluted drinking water, food, and air. Of the 52 participants, 27% (men and women equally represented; 12 from Menoufia and 3 from Giza) specified blood-to-blood contact as the principal mode of transmission, and four of these respondents worked in the healthcare sector. Other causes of HCV mentioned by fewer than three participants included infected people coming from other countries and transmitting the infection to Egyptians, ignorance, lack of health awareness, drug addiction, and sharing personal equipment and contaminated objects.

More than half of all participants (54%) thought that causes of HCV in Egypt were different from those in other countries, and the most common reasons mentioned were: differences in customs, traditions, and cultures (19%); better environmental conditions (31%); and a higher level of development elsewhere (25%). Seven participants commented on a lack of public health authorities’ attention to HCV. Twenty-six participants did not know whether or not there were differences.

When asked about HCV disease manifestations, 45% of the participants stated that they did not know the answer, while the others gave one or more responses. Among the twenty participants who mentioned yellowish discoloration or color changes of the eye, face, and skin, six worked in the health sector. Non-specific general symptoms such as exhaustion and inability to work were mentioned by 13 participants, and gastrointestinal manifestations such as nausea, vomiting or diarrhea were mentioned by 16. Four mentioned flu-like symptoms.

### Perceptions of prevention and treatment of HCV

Two questions in the survey assessed participants' perceptions regarding the actions they, their families, and the community could take to prevent HCV infection in Egypt. Environmental issues such as general cleanliness, safe food, and safe water dominated the responses. Table 2 summarizes the responses. Personal and home cleanliness were the most reported practices followed by clean environment, safe food and drinking water, having regular medical checkups, and not sharing personal equipment. Five participants thought that avoiding HCV was a matter of fate and that God would protect them. In addition, pesticide control, adequate waste handling, early detection and management of disease, and health education were mentioned as potential preventive measures the community could take.

**Table 2:** Perceived roles of individuals, their families, and the community in preventing HCV infection

	**Number (total = 67)**	Percent*
**What you and your family can do**		
Home/personal cleanliness	27	40
Not sharing personal equipment	13	19
Care of oneself/home/family	12	18
Medical check-ups	10	15
Eat safe food	10	15
Drink safe water	4	6
Have faith in Allah	5	8
Don’t know	4	6
**What the community can do**		
Have a clean environment and practice general cleanliness	13	19
Practice food sanitation	5	8
Make medical check-ups available	11	16
Have safe water	7	10
Provide treatment for diseased individuals	7	10
Provide health education	6	9
Take medical precautions	4	6
Have faith in Allah	7	10
Don’t know	9	13

*Percents add to more than 100% due to multiple responses.

Availability of proper treatment was mentioned by six participants as a preventive measure. Two of the six participants who brought up the issue of health education and raising awareness among the Egyptian population specifically mentioned the role of television and mass media in warning people about the disease. Four participants mentioned various medical precautions pertaining to blood transfusion, injections, and syringes. Four people did not specify any measures, but they believed that disease prevention was always better than relying on treatment; four participants thought that prevention and treatment were up to God. One participant believed that HCV prevention and control was a purely governmental responsibility.

When asked if HCV infection can be successfully cured, 39% of participants answered that medication is effective, and 18% indicated that they did not know. Eleven respondents (16%) answered that there is no treatment that cures HCV infection. Participants were also asked about the sources that they thought would be most effective in providing information about HCV to the general population in Egypt (Table 3). The majority of both women (88%) and men (79%) responded that physicians and television, respectively, would be effective information sources. Other sources that were mentioned included billboards and signs, religious leaders, and nurses or other healthcare workers; less common answers included friends, relatives, and printed materials.

**Table 3:** Sources that participants believed to be most effective in providing information to the public on HCV infection

	**Number (total = 67)**	Percent*
Physicians	55	82
TV	52	78
Billboards and signs	24	36
Religious leaders	16	24
Health workers	13	19
Nurses	12	18
Radio	12	18
Newspapers	10	15
Street posters	10	15
Brochures	10	15
Relatives	9	13
Friends	9	13
Booklets	8	12

*Percents add to more than 100% due to multiple responses.

## Discussion

We evaluated the knowledge and perceptions of HCV infection and pesticides use in two communities in rural Egypt. We found that there was a considerable lack of knowledge pertaining to the adverse health effects of pesticides and basic prevention and treatment of HCV infection, all of which are associated with HCC in the Middle East and North Africa (MENA) region. This is of particular concern given that Egypt has the world’s highest prevalence rate of HCV [17,18] and that a large proportion of the population is engaged in agriculture and directly exposed to pesticides [6].

Nearly two-thirds of respondents reported using pesticides in the home, but less than 40% reported having been informed about the health effects of pesticides, and of these, just five individuals reported learning this from a health care provider. This is likely an explanatory factor in the finding that one-fifth of respondents could not name a health effect of pesticides. It is also consistent with the findings of a 2010 study in Egypt, conducted by the World Bank, revealing that in primary care clinics, including those in rural areas, “relatively little attention is paid to prevention of chronic diseases and promotion of health habits” [19]. Another study in Egypt attributed lower knowledge about pesticides and its unsafe use to lower levels of education [20]. In our study, a third of participants never attended school, but all of the pesticide applicators (N = 11) were aware of the risks of pesticides - yet only one used the appropriate protective gear. Our findings are consistent with previous reports of farm workers from various MENA countries having awareness of pesticide toxicity and safety procedures but nonetheless taking part in unsafe behavior at work [21] or experiencing occupationally-related pesticide toxicity symptoms [22,23]. In our study only one agricultural worker out of the 11 pesticide applicators sought care from a pharmacist for a rash. The fact that knowledge of the harmful effects of pesticides does not necessarily equate with proper use of preventive measures is not unique to the MENA region. Indeed, studies in Brazil have revealed similar results; in 2006 Recena and colleagues found that the majority of farm workers knew that pesticides are harmful to human health, but less than 20% were compliant with the use of personal protective equipment [24]. Another study in Brazil from 2012 showed similar results [25]. Therefore, we believe that simple knowledge of the facts is not sufficient for appropriate compliance with safety procedures; better risk communication by trusted healthcare workers or safety officers is paramount for effective implementation of protective measures. Indeed, even among farmers who were well-informed and willing to implement safety procedures, cases of poisoning have been reported [26] due to improper use of protective equipment, which can be uncomfortable to wear and is expensive.

We found that over 45% of the participants did not know about viral hepatitis disease manifestations, and 22% did not know the causes of HCV infection. Among those who knew that it is a virus, 81% mentioned incorrect modes of transmission. Similar studies in Pakistan revealed that 38% of barbers, the majority of whom were uneducated, did not know about hepatitis B and C and their transmission modes [27,28]. A study of hairdressers in Italy revealed that despite the fact that 93% were aware of blood borne pathogens including HCV, 22% failed to sterilize cutting instruments between customers [29]. Such lack of knowledge, across cultures and levels of literacy, is not unique to HCV. In a study of knowledge and attitudes about malaria transmission and prevention in Tanzania, gaps in knowledge about parasite transmission and appropriate preventive measures were found to determine communities’ predispositions to disease epidemics [30]. Here again, risk communication and better health messages are needed. Health education in rural Egypt was reported to be effective in increasing the knowledge of parents of how to protect their children from pesticide exposure, not only in the young and educated, but also to a lesser extend among illiterate individuals [31] and more so with the use of videotape than with lecture. Furthermore, for health promotion and disease prevention initiatives at the community level, empowering members of the community to carry out the task has been reported to be effective in Egypt [32].

When asked what can be done to prevent HCV infection in Egypt, the majority of our participants emphasized the importance of safe water, safe food, and general cleanliness, and less than one fifth mentioned the role of HCV screening or medical check-ups. This could be partially caused by deficient knowledge of HCV transmission risk factors. However, it is consistent with a recent study conducted on a representative sample of Cairo residents showing community ranking of priorities to reduce major health hazards: the majority of participants in that study prioritized improved public water supplies over HCV screening and treatment [33].

The role of religion was not a major factor in the views of participants regarding the actions that families or the community could take with respect to HCV. However, it was notably important in terms of their views on ways that HCV could be treated; just over one-fifth of respondents believed that prevention and treatment of HCV is up to God, whereas less than half this number believed that there is a medical treatment available. The lack of trust in clinicians in public healthcare facilities, reported in both a recent World Bank study of Egyptian health facilities [19] and in a study of youth in South Egypt [34], may contribute to those beliefs.

The most significant limitations of our study are its relatively small sample size and limited geographical scope. This pilot test was not intended to be generalizable but rather to serve as the basis for further development of health education interventions in this region of Egypt.

## Conclusion

In summary, this study revealed that participants from two rural villages in Northern Egypt, residing in a region with one of the world’s highest HCV prevalence rates, were unaware of the major transmission routes for the virus and suffered from profound misconceptions regarding the prevention of HCC. Similar but less severe gaps in knowledge were found regarding the toxicity and prevention of unsafe pesticides exposures. The results of this study may inform improved training of healthcare workers and agricultural extension workers, both of whom could play a greater role in educating the Egyptian population about HCC, its major risk factors, and the need for multiple avenues of prevention.

## Abbreviations

HBV: Hepatitis B virus; HCV: Hepatitis C virus; HCC: Hepatocellular carcinoma; MENA: Middle East and North Africa.

## Competing interests

There are neither any financial competing interests nor any non-financial competing interests (political, personal, religious, ideological, academic, intellectual, commercial or any other) to declare in relation to this manuscript.

## Authors’ contributions

DAS participated in the study design, field work, data analysis, and manuscript writing. SA contributed to study design, data analysis, and manuscript writing. IAJ supported study design, data analysis, and manuscript writing. JW participated in study design and manuscript writing. WAK contributed to field work, data analysis and manuscript writing. CAL supported study design, data analysis and manuscript writing. All authors read and approved the final manuscript.

## Pre-publication history

The pre-publication history for this paper can be accessed here:

http://www.biomedcentral.com/1471-2458/14/501/prepub

## Supplementary Material

Additional file 1 English Questionnaire. Click here for file

Additional file 2 Arabic Questionnaire. Click here for file
