# Supplementary material for: Knowledge and perceptions of hepatitis c infection and pesticides use in two rural villages in Egypt
Source: BMC Public Health. 2014 May 24;14:501. doi: 10.1186/1471-2458-14-501 (PMC4050414; doi:10.1186/1471-2458-14-501)
Supplement: Additional file 1 — English Questionnaire. [file 1471-2458-14-501-S1.docx]

**Questionnaire for HCC prevention study**

**All data and information in this study are highly confidential, and will Not be used except for scientific research.**

**Participant’s number**:

Date of data collection (day/month/year) ……/…../20..

Interviewer’s number:

| **Number** | Question | Answer |
| --- | --- | --- |
| 1 | Gender | 1: Male 2: Female |
| 2 | What is your age | Age in years_______ |
| 3 | When were you born? | Year 19__ |
| 4 | Where were you born? | Governorate: _________  98: Outside Egypt 99: Don’t know |
| 5 | What is the level of education you reached? | 1: Never joined school; 2: Kottab; 3: Literacy classes; 4: Primary education; 5: Preparatory education; 6: Secondary education; 7: Technical and vocational education; 8 Above secondary but less than university; 9: University; 10: Postgraduate studies |
| 6 | Can you read and write? | 1: Reads and writes; 2: Reads only; 3: Reads and writes a little; 4: Does Not know how to read or write; 5: Went to school but does Not know how to read or write |
| 7 | During most of your life, have you worked outside your home? | 0: No; 1: Yes |
| 8 | What was your main job? | ---------------------------------- |
| 9 | Have you had other jobs? | 0: No; 1: Yes |
| 10 | What were these? | --------------------------------------- |

**Now I am going to ask you about some points related to the “liver”:**

| **Number** | Question | Answer |
| --- | --- | --- |
| **11** | Have you ever heard of the “bad disease” that affects the liver, I mean “liver cancer”? | 0: No; 1: Yes |
| **12** | What causes liver cancer? |  |
| **12.1** | 1. Hepatitis B and C viruses | 0: No; 1: Yes; 9: Don’t know |
| **12.2** | 1. Water pollution | 0: No; 1: Yes; 9: Don’t know |
| **12.3** | 1. Air pollution | 0: No; 1: Yes; 9: Don’t know |
| **12.4** | 1. Pesticides | 0: No; 1: Yes; 9: Don’t know |
| **12.5** | 1. Foods contaminated by fungus | 0: No; 1: Yes; 9: Don’t know |
| **13** | Can liver cancer be prevented | 0: No; 1: Yes; 9: Don’t know |
| **14** | How long does it take liver cancer to develop | 1: One or two years; 2: 10 years; 3: 20 or more years; 9: Don’t know |
| **15** | Have you ever heard of HBV and HCV | 0: No; 1: Yes; 9: Don’t know |
| **16** | How are hepatitis B and C viruses spread from one person to another |  |
| **16.1** | 1. Being in contact with sewage or pollution | 0: No; 1: Yes; 9: Don’t know |
| **16.2** | 1. Having contact with infected blood | 0: No; 1: Yes; 9: Don’t know |
| **16.3** | 1. Sharing food or drinks | 0: No; 1: Yes; 9: Don’t know |
| **17** | Can these viruses be detected by a blood test? | 0: No; 1: Yes; 9: Don’t know |
| **18** | Can they be successfully treated by medicines? | 0: No; 1: Yes; 9: Don’t know |
| **19** | Have you ever heard of pesticides? | 0: No; 1: Yes; 9: Don’t know |
| **20** | What are pesticides? |  |
| **20.1** | 1. Chemicals to kill insects | 0: No; 1: Yes; 9: Don’t know |
| **20.2** | 1. Chemicals to kill weeds | 0: No; 1: Yes; 9: Don’t know |
| **20.3** | 1. Chemicals to kill wash clothes | 0: No; 1: Yes; 9: Don’t know |
| **20.4** | 1. Chemicals to kill fungus or mold | 0: No; 1: Yes; 9: Don’t know |
| **20.5** | 1. Chemicals to clean floors | 0: No; 1: Yes; 9: Don’t know |
| **21** | How do people become exposed to pesticides? |  |
| **21.1** | 1. Living near a field that has been sprayed | 0: No; 1: Yes; 9: Don’t know |
| **21.2** | 1. Applying these chemicals in the house | 0: No; 1: Yes; 9: Don’t know |
| **21.3** | 1. Applying these chemicals in farming | 0: No; 1: Yes; 9: Don’t know |
| **21.4** | 1. Eating food that was sprayed in the field | 0: No; 1: Yes; 9: Don’t know |
| **22** | How can people protect themselves from pesticides? |  |
| **22.1** | 1. Handle them according to the directions | 0: No; 1: Yes; 9: Don’t know |
| **22.2** | 1. Get vaccinated against them | 0: No; 1: Yes; 9: Don’t know |
| **22.3** | 1. Avoid going to places that were recently treated | 0: No; 1: Yes; 9: Don’t know |
| **22.4** | 1. Use non-chemical ways to keep pests away | 0: No; 1: Yes; 9: Don’t know |
| **23** | Do you know the fungus that might affect food? | 0: No; 1: Yes; 9: Don’t know |
| **24** | What kinds of foods or drinks are (susceptible) to fungal contamination? |  |
| **24.1** | 1. Cheese | 0: No; 1: Yes; 9: Don’t know |
| **24.2** | 1. Rice | 0: No; 1: Yes; 9: Don’t know |
| **24.3** | 1. Peanuts | 0: No; 1: Yes; 9: Don’t know |
| **24.4** | 1. Oranges | 0: No; 1: Yes; 9: Don’t know |
| **24.5** | 1. Corn | 0: No; 1: Yes; 9: Don’t know |
| **24.6** | 1. Meat | 0: No; 1: Yes; 9: Don’t know |
| **24.7** | 1. Uncooked beans | 0: No; 1: Yes; 9: Don’t know |
| **24.8** | 1. Soda | 0: No; 1: Yes; 9: Don’t know |
| **25** | What can be done to prevent fungus from growing in our food? |  |
| **25.1** | 1. Wash fresh fruits and vegetables when we get them home | 0: No; 1: Yes; 9: Don’t know |
| **25.2** | 1. Store foods off the ground | 0: No; 1: Yes; 9: Don’t know |
| **25.3** | 1. Keep the foods dry | 0: No; 1: Yes; 9: Don’t know |
| **25.4** | 1. Cook foods at a high temperature | 0: No; 1: Yes; 9: Don’t know |
| **26** | Do you believe that you can improve your own health? | 0: No; 1: Yes; 9: Don’t know |
| **27** | Do you believe that you are preventing yourself from getting liver cancer? | 0: No; 1: Yes; 9: Don’t know |
| **28** | Do you plan to make any of the following changes in your life? |  |
| **28.1** | Store food in a way to decrease growth of fungus | 0: No; 1: Yes; 9: Don’t know |
| **28.2** | Use non-chemical ways to keep pests out of the house and away from crops | 0: No; 1: Yes; 9: Don’t know |
| **28.3** | Practice safer ways of handling pesticides | 0: No; 1: Yes; 9: Don’t know |
| **28.4** | Seek medical consultation in case of infection with HBV and HCV | 0: No; 1: Yes; 9: Don’t know |
| **28.5** | Get involved in community efforts to prevent cancer | 0: No; 1: Yes; 9: Don’t know |
