# Supplementary material for: Knowledge and perceptions of hepatitis c infection and pesticides use in two rural villages in Egypt
Source: BMC Public Health. 2014 May 24;14:501. doi: 10.1186/1471-2458-14-501 (PMC4050414; doi:10.1186/1471-2458-14-501)
Supplement: Additional file 2 — Arabic Questionnaire. [file 1471-2458-14-501-S2.doc]

# استمارة بحث عن مكافحة سرطان الكبد

Computer Entry

First Time

Second Time

**جميع البيانات والمعلومات الواردة فى هذه الدراسة سرية للغاية ولن تستخدم إلا لغرض البحث العلمى فقط**

| **مكان الدراسة: ..........................................................................................................................** |  |
| --- | --- |

| **رقم المجيب: ..........................................................................................................** | |  | |
| --- | --- | --- | --- |
|  | | |  |
| **تاريخ جمع البيانات (يوم / شهر / سنة): ................................................................................** | **/ / 200** | | |
|  | | |  |
| **رقم الباحث: .............................................................................................................................** | | |  |

| **الرقم** | **الأســئلة** | الأجـوبة |  | **انتقل إلى** |
| --- | --- | --- | --- | --- |
|  | **اسمك ايه؟ ___________________________** |  |  |  |
| **1** | **النوع:** | **ذكر ............................................**  **أنثى .............................................** | **1**  **2** |  |
| **2** | **عمرك كام سنة؟** | **السن بالسنوات** |  |  |
| **3** | **يا ترى اتولدت / اتولدتى سنة كام؟** | **سنة** | **19** |  |
| **4** | **واتولدت / اتولدتى فين؟** | **محافظة ...................................**  **خارج جمهورية مصر العربية ......................**  **لا يعرف - لا يستطيع التحديد ..................** | **98**  **99** |  |
| **5** | **يا ترى وصلت / وصلتى فى التعليم لحد فين؟** | **لم يدخل مدارس إطلاقا ....................**  **كتاب ....................................**  **فصول محو الأمية ..........................**  **تعليم ابتدائى ..............................**  **تعليم إعدادى .............................**  **تعليم ثانوى ...............................**  **تعليم مهنى وفنى ...........................**  **تعليم فوق المتوسط وأقل من الجامعى .......**  **تعليم جامعى ..............................**  **دراسات عليا .............................** | **1**  **2**  **3**  **4**  **5**  **6**  **7**  **8**  **9**  **10** | **7** |
| **6** | بتعرف / بتعرفي تقرأ وتكتب؟ | **يقرأ ويكتب ..............................**  **يقرأ فقط .................................**  **يقرأ ويكتب قليلا …………………….**  **لا يعرف القراءة والكتابة ..................**  **ذهب للمدرسة ولا يستطيع الكتابة ........** | **1**  **2**  **3**  **4**  **5** |  |
| **7** | **فى أغلب حياتك هل كنت بتشتغل / بتشتغلى شغلة برة البيت؟** | **لأ ............................................... أيوه .............................................** | **صفر**  **1** | **11** |
| **8** | **أيه هى المهنة الرئيسية التى مارستها فى حياتك؟** | **.................................................** |  |  |
| **9** | **فى أى مهن أخرى مارستها فى حياتك؟** | **لأ ............................................... أيوه .............................................** | **صفر**  **1** | **11** |
| **10** | **ايه هى؟** | **................................................** |  |  |

**طيب دلوقثي هأسألك على شوية حاجات تخص الكبد:**

| **الرقم** | **الأســئلة** | الأجـوبة |  | **انتقل إلى** |
| --- | --- | --- | --- | --- |
| **11** | **عمرك سمعت عن المرض الوحش اللي بييجي في الكبد – أقصد سرطان الكبد؟** | **لا ................. صفر أيوه ............. 1** | |  |
| **12** | **طيب تفتكر ايه من الحاجات دي اللي ممكن تسبب سرطان الكبد؟** |  | |  |
|  | **1- العدوى بالفيروسات الكبدية بي وسي .............................** | **لا..... صفر أيوه ..... 1 لا يعرف .....9** | |  |
|  | **2- تلوث المياه ......................................................** | **لا..... صفر أيوه ..... 1 لا يعرف .....9** | |  |
|  | **3- تلوث الهواء .....................................................** | **لا..... صفر أيوه ..... 1 لا يعرف .....9** | |  |
|  | **4- المبيدات ........................................................** | **لا..... صفر أيوه ..... 1 لا يعرف .....9** | |  |
|  | **5- الأكل الملوث بالفطريات أو العفن ................................** | **لا..... صفر أيوه ..... 1 لا يعرف .....9** | |  |
| **13** | **طيب ممكن يبقى فيه وقاية من سرطان الكبد؟** | **لا..... صفر أيوه ..... 1 لا يعرف .....9** | |  |
| **14** | **تفتكر بياخد وقت قد ايه علشان سرطان الكبد يتكون** | سنة أوسنتين .....................................  عشر سنين ......................................  عشرين سنة أو أكثر .............................  لا يعرف ........................................ | **1**  **2**  **3**  **9** |  |
| **15** | **عمرك سمعت عن الفيروسات الكبدية بي وسي؟** | **لا ................. صفر أيوه ............. 1** | |  |
| **16** | **يا ترى ازاي الفيروسات الكبدية بي وسي تتنقل من شخص لأخر؟** |  | |  |
|  | **1-التعرض للمجاري أو التلوث ......................................** | **لا..... صفر أيوه ..... 1 لا يعرف .....9** | |  |
|  | **2- التعرض لدم شخص مريض ......................................** | **لا..... صفر أيوه ..... 1 لا يعرف .....9** | |  |
|  | **3- لما الناس تأكل مع بعض .......................................** | **لا..... صفر أيوه ..... 1 لا يعرف .....9** | |  |
| **17** | **هل يمكن الكشف عن هذه الفيروسات بعمل تحليل دم؟** | **لا..... صفر أيوه ..... 1 لا يعرف .....9** | |  |
| **18** | **هل يمكن الشفاء من هذه الفيروسات؟** | **لا..... صفر أيوه ..... 1 لا يعرف .....9** | |  |
| **19** | **طيب عمرك سمعت عن المبيدات؟** | **لا..... صفر أيوه ..... 1 لا يعرف .....9** | |  |
| **20** | **هأقولك على شوية حاجات وتقولي دي تعتبر مبيدات ولا لأ؟** |  | |  |
|  | 1. **مواد لقتل الحشرات** | **لا..... صفر أيوه ..... 1 لا يعرف .....9** | |  |
|  | 1. **مواد للتخلص من الحشائش الضارة** | **لا..... صفر أيوه ..... 1 لا يعرف .....9** | |  |
|  | 1. **مواد لغسيل الملابس** | **لا..... صفر أيوه ..... 1 لا يعرف .....9** | |  |
|  | 1. **مواد لقتل الفطريات أو العفن** | **لا..... صفر أيوه ..... 1 لا يعرف .....9** | |  |
|  | 1. **مواد لتنظيف الأرضيات** | **لا..... صفر أيوه ..... 1 لا يعرف .....9** | |  |
| **21** | **تفتكر الناس ازاي بيتعرضوا للمبيدات؟** |  | |  |
|  | 1. **السكن جنب الغيطان اللي بتترش** | **لا..... صفر أيوه ..... 1 لا يعرف .....9** | |  |
|  | 1. **استعمال المبيدات في البيت** | **لا..... صفر أيوه ..... 1 لا يعرف .....9** | |  |
|  | 1. **استعمال المبيدات في الزراعة** | **لا..... صفر أيوه ..... 1 لا يعرف .....9** | |  |
|  | 1. **أكل الخضار والفاكهة المرشوشة في الغيط** | **لا..... صفر أيوه ..... 1 لا يعرف .....9** | |  |
| **22** | **طيب ازاي الناس تقدر تحمي نفسها من المبيدات؟** |  | |  |
|  | 1. **استعمالها حسب الارشادات الخاصة** | **لا..... صفر أيوه ..... 1 لا يعرف .....9** | |  |
|  | 1. **أخذ تطعيم للحماية منها** | **لا..... صفر أيوه ..... 1 لا يعرف .....9** | |  |
|  | 1. **متدخلش الاماكن اللي لسه مرشوشةا** | **لا..... صفر أيوه ..... 1 لا يعرف .....9** | |  |
|  | 1. **استعمال حاجات غير المبيدات لابعاد الحشرات** | **لا..... صفر أيوه ..... 1 لا يعرف .....9** | |  |

| **الرقم** | **الأســئلة** | الأجـوبة |  |
| --- | --- | --- | --- |
| **23** | **تعرفي الفطريات أو العفن اللي ممكن ييجي في الأكل؟** | **لا.............صفر أيوه .................... 1** |  |
| **24** | **يا ترى ايه أنواع ألأطعمة والمشروبات اللي ممكن تتلوث بالفطريات أو العفن؟** |  |  |
|  | 1. **الجبنة ...........................................................** | **لا..... صفر أيوه ..... 1 لا يعرف .....9** |  |
|  | 1. **الأرز الني.........................................................** | **لا..... صفر أيوه ..... 1 لا يعرف .....9** |  |
|  | 1. **الفول السوداني ...................................................** | **لا..... صفر أيوه ..... 1 لا يعرف .....9** |  |
|  | 1. **البرتقال ...........................................................** | **لا..... صفر أيوه ..... 1 لا يعرف .....9** |  |
|  | 1. **الذرة ............................................................** | **لا..... صفر أيوه ..... 1 لا يعرف .....9** |  |
|  | 1. **اللحمة النية.......................................................** | **لا..... صفر أيوه ..... 1 لا يعرف .....9** |  |
|  | 1. **الفول الني.........................................................** | **لا..... صفر أيوه ..... 1 لا يعرف .....9** |  |
|  | 1. **الحاجة الصاقعة ..................................................** | **لا..... صفر أيوه ..... 1 لا يعرف .....9** |  |
| **25** | **تفتكر ايه الحاجات اللي ممكن نعملها علشان نمنع الفطريات والعفن انه ييجي في الأكل؟** |  |  |
|  | **1- غسل الخضر والفاكهة عند احضارها الى المنزل .........................** | **لا..... صفر أيوه ..... 1 لا يعرف .....9** |  |
|  | **2- تخزين الأكل والحبوب بعيد عن الأرضيات ..............................** | **لا..... صفر أيوه ..... 1 لا يعرف .....9** |  |
|  | **3- نحفظ الأكل بعيد عن أي رطوبة ....................** | **لا..... صفر أيوه ..... 1 لا يعرف .....9** |  |
|  | **4- نطبخ الأكل في درجات حرارة عالية ...................................** | **لا..... صفر أيوه ..... 1 لا يعرف .....9** |  |
| **26** | **تفتكر انك باذن الله ممكن تحسن صحتك بنفسك؟** | **لا..... صفر أيوه ..... 1 لا يعرف .....9** |  |
| **27** | **تفتكر انك باذن الله ممكن تحمي نفسك انه يجيلك سرطان كبد؟** | **لا..... صفر أيوه ..... 1 لا يعرف .....9** |  |
|  |  |  |  |
| **28** | **هل عندك النية انك تغير حاجة من الحاجات دي في حياتك؟** |  |  |
|  | **1- تخزين الأكل بطريقة تقلل نمو العفن أو الفطريات ......................** | **لا..... صفر أيوه ..... 1 لا يعرف .....9** |  |
|  | **2- تجربة حاجة غير المبيدات لابعاد الحشرات من المنزل ..................** | **لا..... صفر أيوه ..... 1 لا يعرف .....9** |  |
|  | **3- اتباع طرق أكثر أمانا لاستعمال المبيدات ...............................** | **لا..... صفر أيوه ..... 1 لا يعرف .....9** |  |
|  | **4- طلب استشارة طبية في حالة لا قدر الله الاصابة بالفيروسات الكبدية بي أو سي ......................................................................** | **لا..... صفر أيوه ..... 1 لا يعرف .....9** |  |
|  | **5- الاشتراك في الخدمات المجتمعية للوقاية من السرطان ...................** | **لا..... صفر أيوه ..... 1 لا يعرف .....9** |  |

عند انتهاء المقابلة: أشكرك جدا على مشاركتك معنا فى هذا البحث

للعمل المكتبى - مدى جودة البيانات

- تقييم الاستبيان: تم استيفاء البيانات المطلوبة ..................... 1

لم يتم استيفاء البيانات المطلوبة ................. 2

أسباب عدم استيفاء البيانات المطلوبة

...........................................................

...........................................................

1. مراجعة البيانات: رقم القائم بعملية المراجعة:

تاريخ المراجعة : / /
